# Noninvasive Mechanical Ventilation with Average Volume-Assured Pressure Support versus BiPAP S/T in De Novo Hypoxemic Respiratory Failure

**DOI:** 10.1155/2022/4333345

**Published:** 2022-08-03

**Authors:** Killen H. Briones-Claudett, Mónica H. Briones-Claudett, Mariuxi del Pilar Cabrera Baños, Killen H. Briones Zamora, Diana C. Briones Marquez, Luc J. I. Zimmermann, Antonio W. D. Gavilanes, Michelle Grunauer

**Affiliations:** ^1^Universidad de Las Americas, Facultad de Medicina, Quito, Ecuador; ^2^Intensive Care Unit, Ecuadorian Institute of Social Security (IESS), Babahoyo, Ecuador; ^3^Physiology and Respiratory-Center, Ecuador; ^4^Intensive Care Unit, Santa Maria Clinic, Guayaquil, Ecuador; ^5^Universidad Espíritu Santo, Samborondón, Ecuador; ^6^Universidad de Guayaquil, Facultad de Ciencias Médicas, Guayaquil, Ecuador; ^7^School for Oncology and Developmental Biology (GROW), University of Maastricht, Maastricht, Netherlands; ^8^School of Medicine, Universidad San Francisco de Quito, Quito, Ecuador

## Abstract

**Background:**

Bilevel positive airway pressure in spontaneous/time and average volume-assured pressure support (BiPAP·S/T–AVAPS) could maintain an adequate tidal volume by reducing the patient's inspiratory effort; however, this ventilatory strategy has not been compared with other ventilatory modes, especially the conventional BiPAP S/T mode, when noninvasive mechanical ventilation (NIMV) is used. The primary objective of this study was to determine the rate of success and failure of the use of BiPAP·S/T-AVAPS versus BiPAP·S/T alone in patients with mild-to-moderate “de novo” hypoxemic respiratory failure.

**Methods:**

This was a matched-cohort study. Subjects with mild-to-moderate de novo hypoxemic respiratory failure were divided into two groups according to the ventilatory strategy used. The subjects in the BiPAP·S/T group were paired with those in the BiPAP·S/T-AVAPS group.

**Results:**

A total of 58 subjects were studied. Twenty-nine subjects in the BiPAP·S/T group were paired with 29 subjects in the BiPAP·S/T-AVAPS group. Twenty patients (34.5%) presented with “failure of NIMV,” while 38 (65.5%) patients did not. In addition, 13 (22.4%) patients died, while 45 (77.6%) recovered. No differences were found in the percentage of intubation (*P*=0.44) and mortality (*P*=0.1).

**Conclusion:**

The BiPAP S/T-AVAPS ventilator mode was not superior to the BiPAP·S/T mode. A high mortality rate was observed in patients with NIMV failure in both modes. This trial is registered with https://doi.org/10.1186/ISRCTN17904857.

## 1. Background

Noninvasive mechanical ventilation (NIMV) is an effective treatment for pulmonary insufficiency with acute respiratory failure of various etiologies [1, 2]. Regarding the use of NIMV in patients with acute respiratory failure (ARF), continuous positive airway pressure (CPAP) and bilevel positive airway pressure (BiPAP) have traditionally been chosen depending on the clinical condition, underlying disease, and severity of patients [3, 4].

The ventilatory strategy, bilevel positive airway pressure in spontaneous/time and average volume-assured pressure support (BiPAP·S/T-AVAPS), allows the use of a fixed preprogrammed tidal volume (TV), which is kept constant by virtue of inspiratory pressure variations [5]. This ventilatory strategy estimates the delivered volume and adjusts its parameters to ensure a predetermined “target volume” [6]. The initial information about the use of BiPAP·S/T–AVAPS was focused on patients with chronic respiratory diseases, obstructive sleep apnea, or alveolar hypoventilation [7, 8]. However, in our prior study, in case of ARF, a rapid sensory recovery was observed with the early use of this ventilatory strategy in patients with exacerbations of chronic obstructive pulmonary diseases (COPD) [9]. Subsequently, other authors have also observed similar results when BiPAP·S/T–AVAPS was used in patients with exacerbated COPD [10, 11].

We evaluated the BiPAP·S/T–AVAPS mode in patients with de novo hypoxemic respiratory failure and observed that it provides a better approach to protective ventilation in a selected group of subjects with mild-to-moderate hypoxemic respiratory insufficiency and spontaneous breathing to control the exhaled TV and inspired pressures [12].

It is now known that high exhaled TV and inspired airway pressure during NIMV use cause alterations in the bioelasticity of the respiratory system with lung damage. Therefore, to avoid acute lung injury, an optimal ventilatory option is used to limit both the exhaled TV and inspired pressure. At present, this strategy, although described in patients with hypoxemic respiratory failure, has not been compared with other ventilatory modes, especially the conventional mode of BiPAP·S/T when using NIMV.

There is a lack of information and research studies that compare the efficacy of the new ventilatory modes used in NIMV, specifically the ventilatory strategy with BiPAP·S/T-AVAPS, in the management of patients with ARF, making this topic attractive and novel.

This study was designed to compare the results of the use of guaranteed volume support pressure BiPAP·S/T-AVAPS versus BiPAP·S/T alone in patients with mild-to-moderate de novo hypoxemic respiratory failure. The primary objective was to compare the rate of rescue intubation between the two different modes. Secondary outcomes were days of NIMV, ICU stay, hospital stay, and mortality.

## 2. Methods

### 2.1. Study Design

A matched-cohort study was conducted between January 2010 and December 2013 in which all participants were admitted at the Santa Maria Hospital and Military Hospital. The study was approved by the ethics committee of Santa Maria Hospital and Military Hospital (Approval number: N/REFE 01/12/2013, Protocol/serial/number: 2013 (1)). Informed consent was signed by a surrogate if the subject lacked the autonomy necessary for consent.

A total of 58 patients were recruited in this study, and patients were divided into two groups according to the ventilatory strategy used: 29 in the BiPAP·S/T-AVAPS group and 29 in the control group (BiPAP·S/T alone). The person responsible for the decision to participate in the study was the patient or surrogate if he/she was not capable of making the decision. The study was conducted based on CONSORT regulations (https://doi.org/10.1186/ISRCTN17904857).

This was a prospective study; for each patient treated with BiPAP·S/T-AVAPS, one matched control was selected, according to the following matching criteria: cause of ARF, severity of illness on admission within assessment score of acute physiology and chronic health evaluation (APACHE II) 5 points, age difference ≤5 years, ratio of partial arterial oxygen pressure and inspired fraction of oxygen (PaO_2_/FiO_2_) within 10 points of the value of the treated group, and partial pressure of carbon dioxide (pCO_2_) within 5 points.

The matched group was selected from a consecutive database of patients previously treated with BiPAP S/T in our intensive care units (ICUs).

NIMV was administered on the first day for 24 h according to patients' tolerability. When NIMV was suspended, patients received oxygen through a mask connected to an oxygen reservoir having an FIO_2_ of 0.5 to 0.6, for the shortest possible time. NIMV was restored as soon as patients' pulse oximetry SO_2_ reached 90% or less.

### 2.2. Selection of Patients

Patients presented with mild (PaO_2_/FiO_2_, 200–300 mmHg) to moderate (PaO_2_/FiO_2_, 100–200 mmHg) de novo hypoxemic respiratory failure with PaO_2_/FIO_2_ ≤ 300 mmHg were enrolled in this study.

### 2.3. Inclusion Criteria

The patients enrolled in this study met the following criteria: (1) ≥ 18 years of age; (2) admitted to an intensive care unit (ICU); and (3) diagnosed with ARF: inadequate oxygenation (PaO_2_ < 60 mmHg), breathing ambient air (SaO_2_ < 92%), PaO_2_/FiO_2_ < 300 mmHg, and severe dyspnea (RR > 25 breaths/minute), with use of accessory muscles, according to the noninvasive ventilation care standards committee of the British Thoracic Society (British Thoracic Society Standards of Care Committee) [13, 14].

### 2.4. Exclusion Criteria

Participants were excluded based on the following criteria: (1) patients with facial deformity; (2) obstruction of the upper airway by surgery or trauma; (3) alterations in the central nervous system not related to hypercapnic encephalopathy; (4) cardiogenic pulmonary edema, pulmonary embolism, pneumothorax, hemoptysis, or septic shock; (5) urgent intubation due to cardiorespiratory arrest and hemodynamic instability with systolic pressure (SBP) < 80 mmHg; (6) if they demonstrated hemodynamic instability or excess respiratory secretions; (7) if they did not cooperate or were agitated or could not use the device interface; (8) if the patient had recently undergone upper airway surgery; and (9) if the patient had received NIMV with “Do Not Resuscitate” order.

### 2.5. Intervention

Patients assigned to the intervention group received NIMV with guaranteed volume support pressure (BiPAP·S/T-AVAPS) for 12 h a day for at least 24 h.

### 2.6. Ventilatory Strategy in BiPAP·S/T–AVAPS Mode

Ventilation parameters initially programmed in the BiPAP·S/T-AVAPS mode were as follows: maximum inspiratory positive airway pressure (IPAP), 14–26 cmH_2_O; minimum IPAP, 8 cmH_2_O; expiratory positive airway pressure (EPAP), 6–8 cmH_2_O.

The programmed tidal volume (TV) was 6–8 mL/kg of ideal body weight calculated using the following formula: 55.5 ± 2.3 × (height in inches−60) kg for men and 45.5 ± 2.3 × (height in inches−60) kg for women. The respiratory rate (RR) was 14–20 breaths/min, rise time was 300–400 ms, and inspiratory time was 0.8–1.2 s. FiO_2_ was programmed to maintain SaO_2_ above 90%. The maximum IPAP, exhaled TV (ETV), volume per minute (Vmin), and leaks were controlled using a ventilator software. The synchronization of BiPAP with AVAPS was carried out using Auto-Trak (Respironics Inc., Murrysville, Pennsylvania, USA), along with a series of Mirage IV (Resmed) facial masks.

### 2.7. Ventilatory Strategy in BiPAP S/T Mode

Ventilatory parameters initially programmed in the S/T mode were as follows: IPAP, 12–20 cmH_2_O, according to the physician's assessment; EPAP, 6 cmH_2_O; rRR, 15 breaths/min; rise time, 300–400 ms; and inspiratory time, 0.6–1.2 s. The IPAP was progressively augmented with 2 cmH_2_O, according to the prescription of the attending physician. Oxygen supplements were added through an O_2_ adapter close to the mask to maintain the SaO_2_ above 90%. The exhaled current volume (EVT), Vmin, and leakage were controlled using a ventilator software. The synchronization of BiPAP with AVAPS was carried out using Auto-Trak (Respironics Inc. Murrysville. Pennsylvania. USA), a series of Mirage IV (Resmed) masks, and a Confourt Series II mask (Respironics).

### 2.8. Measurements

Arterial blood gases (ABG) were measured at baseline and 1, 12, and 24 h after the use of NIMV. Patients were evaluated by a respiratory therapist under strict supervision of the physician trained in NIMV. Complications of mask use were reported, if any. The severity of the disease was assessed by the APACHE II score. Maximum programmed VT, maximum patient IPAP, exhaled tidal volume, Vmin, leakage, RR of the patient, and IPAP were reported at baseline and during the first 48 h of NIMV obtained in the early morning hours. The mask use and tolerability were also evaluated. The tolerance capacity of the mask (comfort score) was evaluated as follows: 1 = low, 2 = average, and 3 = good [15]. Complications inherent to the use of the technique, including abdominal distension, skin necrosis, epistaxis, and ear pain, were also evaluated [16].

### 2.9. Discontinuation of NIMV

NIMV was initially used continuously in accordance with the patients' tolerance until the PaO_2_/FiO_2_ ratio was above 400 mmHg, and the decision of the medical staff considered a partial or total resolution of the cause that led to receiving NIMV. The weaning process was initiated when clinical stability was achieved, which was defined as an RR less than 25 resp/min, a heart rate (HR) of 100 beats/minute, improvement in the level of consciousness, compensation of the pH, ambient air with SaO_2_ > 90%, or a low rate of FiO2 (3 L/min). NIMV was discontinued when the patient remained stable for >24 hours.

### 2.10. Comparisons

Comparisons were made between the two groups (BiPAP·S/T-AVAPS and BiPAP·S/T). Patients were classified according to “de novo” hypoxemic respiratory failure, from mild to moderate.

The following variables were evaluated between groups: age, sex, weight in kg (PBW), APACHE II, risk factors for acute respiratory distress syndrome (ARDS), community pneumonia, postoperative acute abdomen, acute pancreatitis, involvement of radiographic quadrants, SBP (mmHg), diastolic blood pressure (DBP) (mmHg), HR (beat/min), RR (breaths/min), PaO_2_/FiO_2_ ratio, ABGs including pH carbon dioxide concentration (pCO_2_) (mmHg), partial pressure of arterial oxygen (PO_2_) (mmHg), blood bicarbonate (HCO_3_) (mmol/L), excess base (EB), FIO_2_ (%), ventilation mode (spontaneous/time), IPAP (cmH_2_O), EPAP (cmH_2_O), inspiratory time (IT) (mseg), ramp, ETV (mL), Vmin (L/min), leakage (cmH_2_O), radiological involvement (one quadrant, two quadrants, three quadrants, four quadrants), days of NIMV, days of hospital stay, intubation, and mortality.

### 2.11. Outcome Measures

The primary outcome was success or failure of NIMV (requirement for endotracheal intubation). The secondary outcomes were ICU duration of hospitalization and mortality.

### 2.12. Calculation of Sample Size

In this study, to detect a 20% difference between success and failure of NIMV between the two groups with a Type I error of 0.05 (Alpha significant) and a Type II error of 0.20 (Beta, 1-power), sixty patients (30 in each group) were required.

### 2.13. Statistical Analysis

Statistical analyses were performed using MedCalc Statistical Software, version 16.4.3 (MedCalc Software bvba, Ostend, Belgium; https://www.medcalc.org, 2016).

All data are expressed as mean ± standard deviation (SD) for continuous variables and percentages for categorical variables. Normally distributed continuous variables were determined using the Kolmogorov-Smirnov test and were compared using Student's *t*-test. For nonparametric distribution variables, the chi-square or Fisher's exact test was used, depending on the case. The NIMV results were compared between the two groups. Statistical significance was set at *P* value < 0.05.

## 3. Results

**Figure 1:**
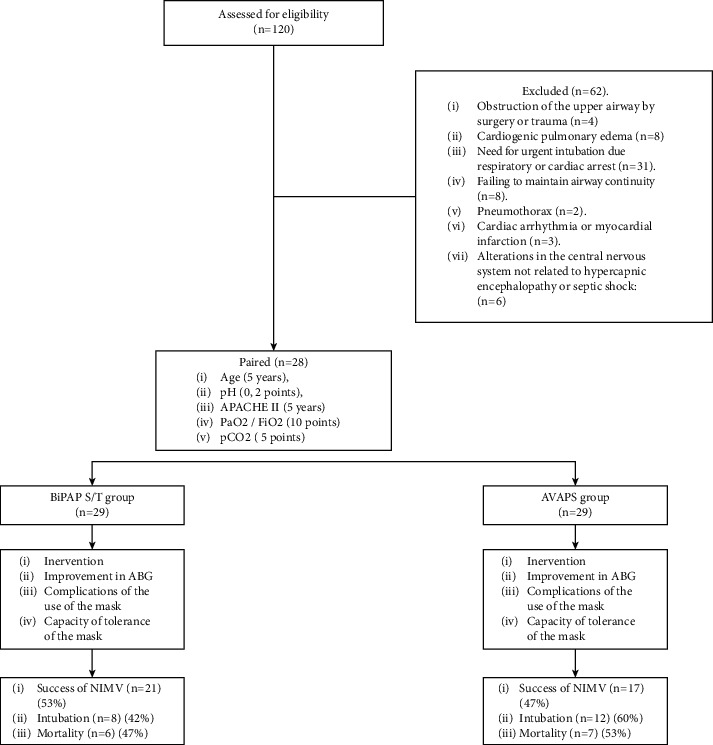
Flow chart of selection of patients.

A total of 58 subjects were studied (29 subjects in the BiPAP S/T group were paired with 29 subjects in the BiPAP·S/T-AVAPS group) (Figure 1). The average age of the study population was 68.7 ± 19.5 years; the average hospitalization days were 11.8 ± 6.3; the average days of stay in ICU were 7.9 ± 4.9; the average days that subjects received NIMV were 4.3 ± 1.79.

The ABG levels were as follows: pH, 7.43 ± 0.1; pCO_2_, 32.5 ± 5.8 mmHg; PO_2_, 74.9 ± 9.6 mmHg; HCO_3_, 22.6 ± 4.1 mmol/L; SaO_2_, 94% ± 4% during the first 10 min of the initial ventilatory support.

The vital signs of the patients were as follows: SBP, 123.3 ± 19.4 mmHg; DBP, 74.3 ± 8.7 mmHg; HR, 97.6 ± 13.7 beats/min; RR, 29.3 ± 4.7 breaths/min.

The ventilatory parameters included the following: VT-AVAPS levels, 489.7 ± 74.5 ml; maximum IPAP levels, 17 ± 4.04 cmH_2_O; patient IPAP levels, 15 ± 3.2 cmH_2_O; leaks, 17.1 ± 9 cmH_2_O, ETV, 473.3 ± 196.1 mL, Vmin, 10.8 ± 4.15 L/min. The baseline characteristics are presented in Table 1.

No differences were found between the BiPAP·S/T and BiPAP·S/T–AVAPS groups (Table 2).

In the study, 20 patients (34.5%) presented with “failure of NIMV,” while 38 (65.5%) patients did not. In addition, 13 (22.4%) patients died, while 45 (77.6%) recovered. No differences were found in the percentage of intubation (*P*=0.44) and mortality (*P*=0.1) (Table 3).

The physiological and ventilator parameters in hypoxemic ARF from baseline to 1, 6, and 12 h on BIPAP-ST-AVAPS are presented in Table 4.

## 4. Discussion

In this study, we found no differences in the outcomes of BiPAP·S/T-AVAPS versus BiPAP·S/T alone in patients with “de novo” hypoxemic respiratory failure. There were no differences in the percentage of intubation or mortality in either group, or in the duration of IMV, hospital stay, or ICU stay. To our knowledge, this study is the first to compare the two ventilatory strategies in this subgroup of patients with de novo hypoxemic respiratory failure.

BiPAP·S/T-AVAPS has been shown to be useful in patients with hypercapnic ARF, especially in obstructive pulmonary disease and alveolar hypoventilation [17]. Other studies have shown the advantages of this method in patients with hypercapnia, especially by guaranteeing TV and minute ventilation and decreasing the percentage of intubation compared with conventional BIPAP·S/T [9, 11]. Additionally, AVAPS facilitates successful extubation in ARDS [18].

Current therapeutic options for hypoxemic ARF are limited and mainly focus on minimizing ventilator-induced lung injury (VILI) [19]. It is now known that it is difficult to maintain a low expiratory TV in patients who are receiving NIMV for hypoxemic ARF, which is generally associated with NIMV failure [20]. Studies have shown that high TV is a predictor of NIMV failure, especially in patients with ARF and moderate-to-severe hypoxemia [21].

High inspired pressures with high ETVs were observed in most patients in whom the AVAPS ventilatory strategy failed. In addition, the use of low TVs with limited airway pressure is essential for lung protection. In some cases, especially in patients with hypoxemic respiratory failure, an excessive increase in inspired pressure could increase intrathoracic pressure and reduce venous return. Moreover, the cardiac index decreases at high inspired pressures [22].

AVAPS attempts to maintain an adequate TV by decreasing the inspiratory effort of the patient and increasing the programmed pressure automatically without exceeding the programmed maximum IPAP when the preset TV is not reached [23]. We found a high mortality rate in patients in whom NIMV failed. Furthermore, an increase in mortality due to delayed intubation in patients with NIMV is well documented [24]. However, we did not observe differences in the failure of NIMV between the BiPAP·S/T-AVAPS and BiPAP·S/T groups.

Our study had the following limitations: (1) it is a “single-center” study and not a randomized controlled trial; (2) other types of interfaces used in de novo respiratory failure NIMV, such as helmet systems that tolerate high levels of positive end-expiratory pressure, have not been evaluated; (3) the majority of these patients were diagnosed with community-acquired pneumonia; therefore, the results could not be extrapolated to hypoxemic respiratory failure of another etiology; (4) the effect of the learning curve could influence the results because our results were obtained in two ICUs with staff having extensive NIMV experience and familiarity with the use of the AVAPS ventilatory strategy, which should be considered when generalizing the results to other centers with less experience.

Nevertheless, we believe that this study provides important data, as this is the first study to evaluate these two ventilatory modes of BiPAP·S/T-AVAPS vs. BiPAP·S/T alone in patients with “de novo” hypoxemic respiratory failure. However, a large-scale randomized controlled study is necessary to assess and compare this approach with other strategies such as continuous high-flow nasal oxygen therapy [25].

## 5. Conclusion

The results of this study suggest that BiPAP·S/T-AVAPS can be used in patients with ARF. Patients with hypercapnic ARF had a greater positive response to a ventilation strategy with BiPAP·S/T-AVAPS than those with de novo hypoxemic ARF. BiPAP·S/T-AVAPS mode ventilators were not superior to the conventional NIMV mode BiPAP·S/T. A high percentage of mortality was observed in patients with failure of NIMV in both modes, especially “de novo” respiratory hypoxemic failure.

## Figures and Tables

**Table 1:** Baseline characteristics of the study population.

Patient characteristics (*n* = 58)	
Variables	Mean
Age (years), mean (SD)	68.3 ± 19.3
Sex, *N* (%)	39 (67) men and 19 (32.8) women
Weight, kg (PBW), mean (SD)	65.5 ± 10
APACHE II score, mean (SD)	18.7 ± 3.0
Risk factors for ARDS	
Community pneumonia, *N* (%)	(87.9) (%)
Postoperative acute abdomen, *N* (%)	(1.7) (%)
Acute pancreatitis, *N* (%)	(10.3) (%)
Clinical and radiographic parameters	
SBP, mmHg, mean (SD)	123.7 ± 19.1
DBP, mmHg, mean (SD)	74.7 ± 8.8
HR, beat/min, mean (SD)	97.7 ± 13.6
RR, breath/min, mean (SD)	26.6 ± 6.6
Radiographic quadrants affected, number, mean (SD)	1.5 ± 0.6
Gasometric parameters	
PAFI (BASAL), mean (SD)	183.5 ± 31.5
pH, mean (SD)	7.43 ± 0.06
pCO_2_, mmHg, mean (SD)	32.5 ± 5.8
PO_2_, mmHg, mean (SD)	75.3 ± 9.6
HCO_3_, mmol/L, mean (SD)	22.5 ± 4.1
EB, mmol/L, mean (SD)	-1.8 ± 6.5
SAT 02 (%), mean (SD)	93 ± 3.1
FiO_2_. (%), mean (SD)	41.6 ± 4.2
Ventilatory parameters	
VT-AVAPS PROGRAMMED, mean (SD)^*∗*^	489.6 ± 54.3
VT, mL/Kg (PBW), mean (SD)	7.0 ± 1
IPAP maximum programmed (AVAPS), cmH_2_O, mean (SD)	15.7 ± 3.2
IPAP patients, cmH_2_O, mean (SD)	14.5 ± 2.8
EPAP, cmH_2_O, mean (SD)	6 ± 0.4
Leakage, cmH_2_O, mean (SD)	17.1 ± 8.9
Vtexh, mL, mean (SD)	473.2 ± 196.1
Vmin, L/min, mean (SD)	13.2 ± 2.5
Final events	
Days of hospital stay, mean (SD)	11.8 ± 6.3
Days of stay in ICU, mean (SD)	7.9 ± 4.9
Days of NIMV, mean (SD)	4.3 ± 1.7
Intubated, *N* (%)	20 (34.5)
Successes of NIMV, *N* (%)	38 (65.5)
Dead, *N* (%)	13 (22.4)

APACHE II, assessment score of acute physiology and chronic health evaluation; SBP: systolic blood pressure; DBP: diastolic blood pressure; HR: heart rate; RR: respiratory rate; PAFI: ratio of partial arterial oxygen pressure and inspired fraction of oxygen; PCO_2_: partial pressure of carbon dioxide; HCO_3_: bicarbonates; EB: excess base; SAT 02: arterial oxygen saturation; FiO_2_: inspired fraction of oxygen; VT-AVAPS: tidal volume average volume-assured pressure support; IPAP: inspiratory positive airway pressure; EPAP: expiratory positive airway pressure; Vmin: volume per minute; Vtexh: exhaled tidal volume; Vt: tidal volume.

**Table 2:** Comparison of characteristics between the groups.

Variables	BIPAP S/T (*n* = 29)	BIPAP S/T-AVAPS (*n* = 29)	*P* value
Age (years), mean (SD)	71.8 ± 18.4	66.9 ± 20.2	0.33
Sex, *N* (%)	Men 20 (69) and women 9 (31)	Men 19 (65.5) and women 10 (34.5)	0.78
Weight kg (PBW), mean (SD)	65 ± 9.6	66.1 ± 10.5	0.67
APACHE II score, mean (SD)	18.4 ± 3.1	19 ± 2.9	0.44
Risk factors for ARDS			
Community pneumonia, *N* (%)	27 (93.1)	24 (82.9)	0.39
Postoperative acute abdomen, *N* (%)	—	1 (3.4) (%)	
Acute pancreatitis, *N* (%)	2 (6.9)	4 (13.8)	
Radiographic quadrants affected, *N* (%)	1.5 ± 0.6	1.5 ± 0.6	1
SBP, mmHg, mean (SD)	75.2 ± 8.8	74.3 ± 8.9	0.7
DBP, mmHg, mean (SD)	125.5 ± 18.5	121.9 ± 19.8	0.47
HR, beat/min, mean (SD)	97.8 ± 14	97.5 ± 13.4	0.93
RR, breath/min, mean (SD)	26.8 ± 7.9	26.4 ± 5.2	0.8
ABGs			
PaO_2_/FiO_2_ (initial), mean (SD)	185.5 ± 30.6	181.6 ± 32.8	0.64
pH, mean (SD)	7.42 ± 0.05	7.43 ± 0.06	0.49
PCO_2_, mmHg, mean (SD)	33.1 ± 6.1	32 ± 5.4	0.47
pO_2_, mmHg, mean (SD)	75.4 ± 8.7	75.1 ± 10.6	0.9
HCO_3_, mmol/L, mean (SD)	22.5 ± 4	22.5 ± 4.3	1
EB, mmol/L, mean (SD)	−2.5 ± 4.7	−1.1 ± 7.8	0.40
SAT 02, (%), mean (SD)	0.93 ± 0.03	0.93 ± 0.03	1
FiO_2_. (%), mean (SD)	0.41 ± 0.03	0.41 ± 0.03	1
Ventilatory parameters			
IPAP, cmH_2_O, mean (SD)	12.8 ± 1.4	16.2 ± 2.8	<0.0001
EPAP, cmH_2_O, mean (SD)	6 ± 0.5	6 ± 0.2	1
RAMP, sec, mean (SD)	3.1 ± 0.4	3.2 ± 0.4	0.3452
IT, sec, mean (SD)	0.94 ± 0.03	0.94 ± 0.03	1
Leakage, cmH_2_O, mean (SD)	17.2 ± 8.7	16.7 ± 9.2	0.83
Vmin, L/min, mean (SD)	13.6 ± 2.5	12.8 ± 2.4	0.21
Vtexh, ml, mean (SD)	456 ± 70.1	453.9 ± 76.4	0.91
Vt/ml/kg (PBW), mean (SD)	7 ± 1	6.9 ± 0.9	0.69

*P* < 0.0001 (statistically significant). APACHE II, assessment score of acute physiology and chronic health evaluation; SBP: systolic blood pressure; DBP: diastolic blood pressure; HR: heart rate; RR: respiratory rate; PaO_2_/FiO_2_: ratio of partial arterial oxygen pressure and inspired fraction of oxygen; PCO_2_: partial pressure of carbon dioxide; HCO_3_: bicarbonates; EB: excess base, SAT 02: arterial oxygen saturation; FiO_2_: inspired fraction of oxygen; IPAP: inspiratory positive airway pressure; EPAP: expiratory positive airway pressure; RAMP: time to change from the expiratory pressure setting to the inspiratory pressure setting; IT: inspiratory time; Vmin: volume per minute; Vtexh: exhaled tidal volume; Vt: tidal volume.

**Table 3:** Comparative outcome in the BiPAP S/T and BiPAP S/*T* + AVAPS groups.

Variables	BIPAP S/T (*n* = 29)	BIPAP S/T-AVAPS (*n* = 29)	*P* value
Days of hospital stay, mean (SD)	11.2 ± 6.5	12.4 ± 6.2	0.47
Days of stay in the ICU, mean (SD)	7.9 ± 5.1	7.9 ± 4.8	1
Days of NIMV, mean (SD)	4.8 ± 1.9	4.1 ± 1.6	0.13
Intubation, *N* (%)	8 (42%)	12 (60%)	0.44
Success of NIMV, *N* (%)	21 (53%)	17 (47%)	0.71
Mortality, *N* (%)	6 (47%)	7 (53%)	0.1

*P* < 0.0001 (statistically significant).

**Table 4:** Physiological and ventilator parameters in hypoxemic ARF from baseline to 1, 6, and 12 h on BIPAP-ST-AVAPS.

pH (mmhg), means ± SD	pH (Baseline)	pH (1 Hour)	pH (6 Hours)	pH (12 Hours)	Value *P*
Failure	7.38 ± 0.05	7.40 ± 0.05	7.41 ± 0.04	7.40 ± 0.05	0.005^*∗*^
Success	7.44 ± 0.05	7.46 ± 0.06	7.45 ± 0.04	7.46 ± 0.02	
pCO_2_ (mmhg), means ± SD	Pco_2_ (Baseline)	Pco_2_ (1 Hour)	Pco_2_ (6 Hours)	Pco_2_ (12 Hours)	Value *P*
Failure	33.8 ± 5.2	33.4 ± 5.0	34.1 ± 5.8	32.3 ± 4.8	0.33
Success	30.2 ± 5.4	31.8 ± 4.5	30.6 ± 5.4	33.3 ± 5.8	
pO_2_ (mmhg), means ± SD	Po_2_ (Baseline)	Po_2_ (1 Hour)	Po_2_ (6 Hours)	Po_2_ (12 Hours)	Value *P*
Failure	77.5 ± 9.9	87.6 ± 20.6	99.4 ± 26.9	106.3 ± 41.2	0.001^*∗*^
Success	75.3 ± 12.5	104.9 ± 32.1	117.5 ± 34.1	126.6 ± 35	
HCO_3_, (mmol/L) means ± SD	HCO_3_ (Baseline)	HCO_3_ (1 Hour)	HCO_3_ (6 Hours)	HCO_3_ (12 Hours)	Value *P*
Failure	22.5 ± 4.4	23 ± 5.5	24.5 ± 7.1	20.8 ± 5.2	0.2
Success	21.6 ± 4.4	23.3 ± 5.1	23.6 ± 4.4	25.7 ± 3.5	
FiO_2_ (%), means ± SD	Fio_2_ (Baseline)	Fio_2_ (1 Hour)	Fio_2_ (6 Hours)	Fio_2_ (12 Hours)	Value *P*
Failure	0.42 ± 0.04	0.44 ± 0.08	0.52 ± 0.09	0.53 ± 0.10	0.001^*∗*^
Success	0.42 ± 0.04	0.39 ± 0.04	0.36 ± 0.06	0.34 ± 0.04	
PaO_2_/FiO_2_ (mmHg)	Pao_2_/Fio_2_ (Baseline)	Pao_2_/Fio_2_ (1 Hour)	Pao_2_/Fio_2_ (6 Hours)	Pao_2_/Fio_2_ (12 Hours)	Value *P*
Failure	185.3 ± 32.8	204.1 ± 67.2	197.0 ± 7 8.6	219.6 ± 148.4	0.001^*∗*^
Success	180.2 ± 35	269.2 ± 87.9	327.9 ± 106.1	340.4 ± 95.5	

^*∗*^ *P* = statistical significance. PCO_2_: partial pressure of carbon dioxide; PO_2_: partial pressure of arterial oxygen; HCO_3_: bicarbonates; FiO_2_: inspired fraction of oxygen; PaO_2_/FiO_2_: ratio of partial arterial oxygen pressure and inspired fraction of oxygen.

## Data Availability

The datasets used and/or analyzed during the current study are available from the corresponding author upon reasonable request.

## ABBREVIATIONS

abrARF: Acute respiratory failure
S: Spontaneous
S/T: Spontaneous/time
BiPAP S/T-AVAPS: Bi-level positive airway pressure in spontaneous/time and guaranteed support pressure with medium volume
BiPAP S/T: Bi-level positive airway pressure in spontaneous/time
PaO2/FiO2: Ratio of partial arterial oxygen pressure and inspired fraction of oxygen
FiO2: Inspired fraction of oxygen
EVT: Exhaled tidal volume
Vmin: Volume per minute
TV: Tidal volume
ARDS: Acute respiratory distress syndrome
SBP: Systolic blood pressure
DBP: Diastolic blood pressure
HR: Heart rate
RR: Respiratory rate
ABGs: Arterial blood gas
pH: The negative algorithm of the hydrogen ion concentration
SaO2: Arterial oxygen saturation
PO2: Partial pressure of arterial oxygen
PCO2: Partial pressure of carbon dioxide
EB: Excess base
IPAP: Inspiratory positive airway pressure
EPAP: Expiratory positive airway pressure
APACHE II: Assessment score of acute physiology and chronic health evaluation
SD: Standard deviation
COPD: Chronic obstructive pulmonary disease
NIMV: Noninvasive mechanical ventilation
IT: Inspiratory time
RAMP: Time to change from the expiratory pressure to the inspiratory pressure setting.
